# Evolution of Pacemakers and Implantable Cardioverter Defibrillators (ICDs) in Cardiology

**DOI:** 10.7759/cureus.46389

**Published:** 2023-10-02

**Authors:** Palash Sahu, Sourya Acharya, Manisha Totade

**Affiliations:** 1 Department of Medicine, Jawaharlal Nehru Medical College, Datta Meghe Institute of Higher Education & Research (Deemed to be University), Wardha, IND

**Keywords:** pulse generator, cardiac resynchronization therapy, pacing, implanted cardioverter defibrillators, pacemakers

## Abstract

Pacemakers and implantable cardioverter defibrillators (ICDs) have revolutionized cardiology by providing life-saving interventions for patients with cardiac rhythm disturbances. Pacing the heart is an effective treatment for people suffering from bradycardia caused by sinus node dysfunction or atrioventricular (AV) block, and electronic pacing has saved countless lives since its introduction into clinical practice. AV synchronization is the typical cycle of atrial depolarization and contraction followed by ventricular depolarization and contraction. The continuation of this cycle leads to appropriate ventricular filling and cardiac output. By contrast, the failure of the cycle results in AV asynchrony, which may result in heart failure. Cardiac resynchronization treatment (CRT) involves using customized pacemakers with or without implantable cardioverter defibrillators and tries to resynchronize the failing heart by enhancing myocardial contraction without increasing energy consumption. This review delves into the extensive journey of pacemakers and ICDs in the field of cardiology. It highlights the transformative impact of these devices on patient care and quality of life, emphasizing technological advancements, clinical applications, and prospects. This comprehensive review aims to provide insights into the dynamic landscape of cardiac rhythm management.

## Introduction and background

Pacemakers, along with implanted cardioverter defibrillators (ICDs), are in use to manage heart rhythms in a broad spectrum of patients with cardiac disorders, such as atrial fibrillation, atrial flutter, and paroxysmal supraventricular tachycardia, and bradyarrhythmia conditions, such as sinus bradycardia and first- and second-degree AV block. At present, they have a specific position in heart failure care, including preventive use of ICDs in patients of severe systolic dysfunction [1]. Both pacemakers and ICDs come under the broad heading of cardiac implantable electronic devices, including biventricular pacemakers and cardiac loop recorders [2].

Directives have been released by the American College of Cardiology, European Society of Cardiology, American Heart Association, European Heart Rhythm Association, and Heart Rhythm Society to determine whether a device is required [3]. As per the directives, for Class I situations, it is widely accepted that a pacemaker should be used; in Class II, patients have symptoms of bradycardia but no evident relationship between symptoms and bradycardia where pacing is unnecessary; in Class III, conditions do not need pacing, such as sinus node disease without symptoms [3].

**Figure 1:**
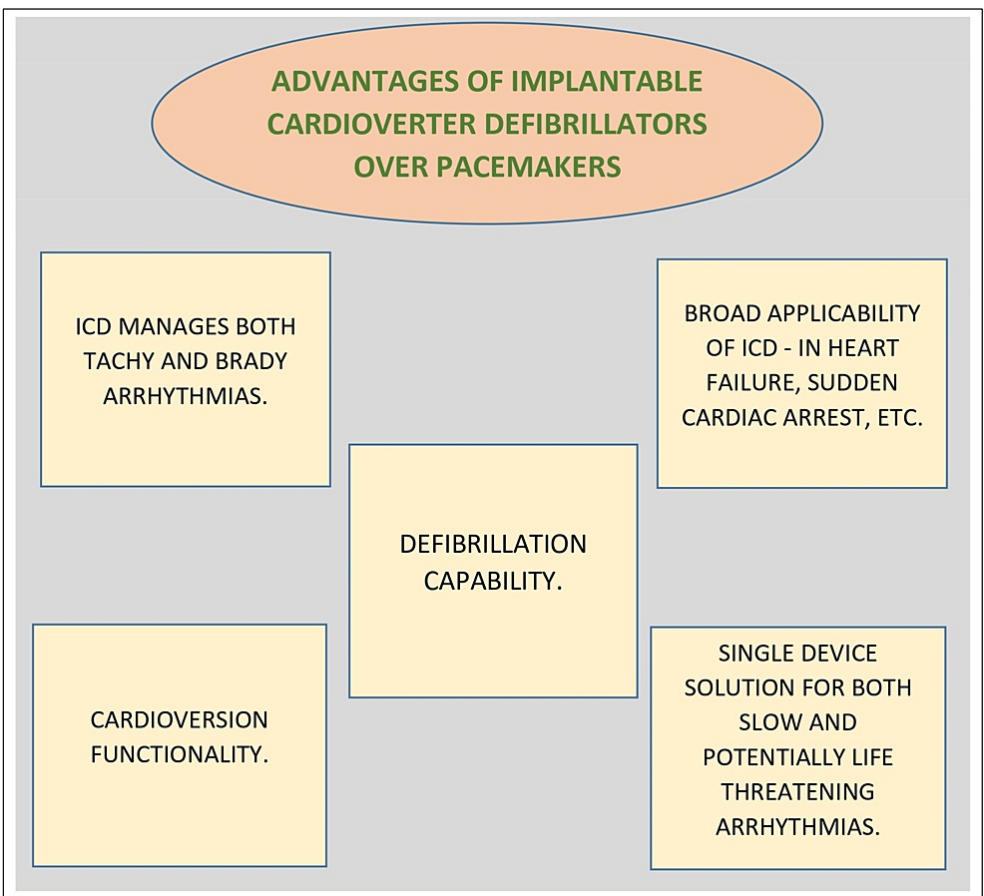
Advantages of implantable cardioverter defibrillators over pacemakers Image created by the authors

Most of the heart failure devices used in clinical practice or currently being researched fall into one of four categories: devices that can monitor heart failure conditions, devices that can manage rhythm abnormalities, gadgets designed to increase the mechanical ability of the heart, and devices to replace a part or all of the heart's function [4]. ICDs and pacemakers are gadgets that can improve the mechanical efficacy of the cardiac system. At the same time, pacemakers address bradycardia (slow heart rhythms). Nonetheless, a new challenge has emerged in the form of unexpected cardiac mortality resulting from ventricular arrhythmias, and the solution came up in the form of ICDs. These devices, introduced in the 1980s [5], provided pacing and incorporated defibrillation capabilities. ICDs could detect life-threatening arrhythmias and deliver high-energy shocks to restore normal rhythms, effectively preventing sudden cardiac death [6]. Figure 1 highlights some essential advantages of ICDs over pacemakers.

The FDA has approved left ventricular assist devices for preserving the circulation of individuals with last stage of heart failure pending cardiac transplantation; they are rapidly being recognized as a viable option to biologic cardiac substitutes [7]. With the advancement of technology, leadless pacemakers are now available in the market [8]. Microelectronics and battery technology have resulted in the miniaturization of pacemakers and ICDs, resulting in smaller device sizes and less invasive insertion procedures. Transitioning from traditional wired to wireless communication between devices increases patient safety and comfort [9,6].

## Review

Methodology

**Figure 2:**
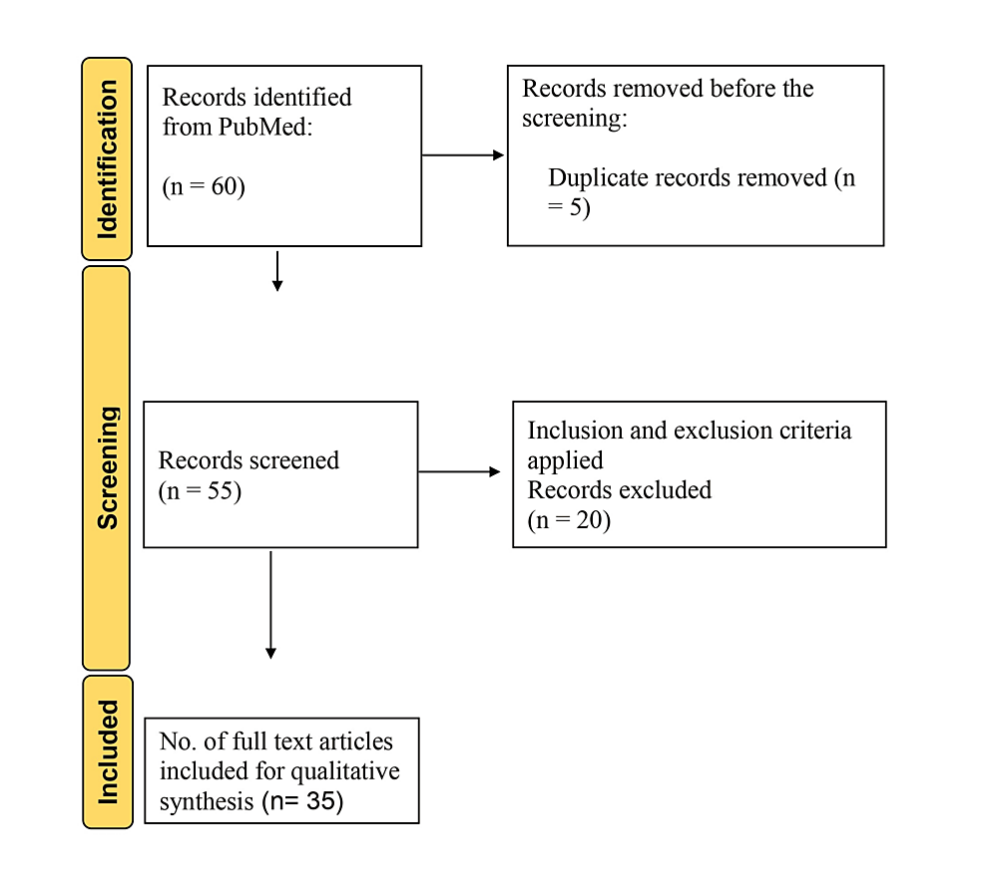
Flowchart of the methodology used for the review Image created by the authors

An English literature search was undertaken using the Internet databases PubMed and Google Scholar with the keywords "cardiology," "pacemakers," "implantable cardioverter defibrillators," "historical background," and relevant synonyms. The search covered papers published from the databases' inception without explicit date constraints. Searching numerous databases, creating inclusion and exclusion criteria, screening papers, and choosing the final research for the review were all parts of the procedure. Peer-reviewed articles published in English focusing on pacemakers and ICDs were included in this article. By contrast, paid articles, articles not in English, and articles not directly related to the topic were excluded. The initial screening consisted of reading the headings and abstracts of the identified papers following the inclusion and exclusion criteria. Full-text articles for possibly relevant research were retrieved, and further screening was performed to pick the final articles for the review. The inclusion criteria were satisfied by 35 papers included in the final review. The search methodology by the PRISMA method is shown in the flow diagram (Figure 2).

Historical background of pacemakers and ICDs

The initial trials at pacing with an implantable pacemaker were made in Sweden in 1958. Before that, thoracic transcutaneous electrodes were used for cardiac stimulation with stainless-steel suture wire as an electrode because external wires posed a risk of infection. Dr. Senning developed a pacemaker and implanted it into Arne Larson, which barely lasted three hours [10]. Initially, the pacemaker was used to treat bradycardia. With technological advancements, pacemakers are currently in use to manage tachycardias, those at prospect of abrupt cardiac death (implantable defibrillators), and people with heart failure (cardiac resynchronization gadgets). The turning point came in the 1960s with the introduction of the first implantable pacemaker. Dr. Wilson Greatbatch's invention of a minor, battery-powered gadget allowed cardiac patients to reclaim their quality of life outside the hospital. The initial designs were primitive compared to today's standards, yet they marked a significant leap forward [11].

Further advancements occurred in pacemakers when implantable cardioverter defibrillators came into the picture, which is the modified version of pacemakers and can function in both brady- and tachyarrhythmia [12]. Dr. Michel Mirowski and his colleagues implanted a patient's first ICD in 1980. In the beginning of the ICD evolution, ICD treatment was not generally acknowledged, and many specialists thought it was unethical. Thus, it took four more years to implant the next ICD in the Netherlands in 1984 at the University Medical Centre Utrecht [13].

Modern pacemakers and ICDs are not only technologically advanced but also highly personalized. These devices can adapt to an individual's activity levels, heart rate variability, and physiological needs. Moreover, they often feature remote monitoring capabilities, enabling healthcare professionals to assess patients' cardiac health remotely and intervene proactively when necessary [14].

Pacemakers

Pacemakers are factitious electrical pulse generators that can create pulses with lengths ranging from 0.5 to 25 milliseconds and output voltages ranging from 0.1 to 15 volts up to 300 times per minute. Even if the device is temporary or permanent, the cardiac specialist or pacemaker technician can examine and regulate the pacing rate, voltage, and pulse width [15]. The classification of pacemakers is based on their functionality and programmability. Single-chamber pacemakers stimulate the atrium or ventricle, while double-chamber pacemakers coordinate both chamber activities for a more natural heartbeat. A cardiac resynchronization therapy (CRT) pacemaker, also called a biventricular pacemaker, was designed for heart failure patients to optimize ventricular synchrony [16]. Double-chamber or single-chamber atrial pacing is considered advanced over single-chamber ventricular pacing because it relates cardiology more intently by supporting atrioventricular (AV) synchrony and sinus node dominance, which can decrease cardiac morbidity and mortality, leading to patient viability and quality of life [17]. Table 1 enumerates some key differences between single- and multi-chamber pacemakers.

**Table 1:** Differences between single- and multi-chamber pacemakers.

Single-chamber pacemaker	Multi-chamber pacemaker
It functions on a single heart chamber.	It can operate on two chambers at the same time.
It is commonly used.	It is less commonly used.
It is cheaper in comparison to a multi-chamber pacemaker.	It is not cost-effective.
Its morbidity and mortality rates are slightly high.	It reduces cardiovascular morbidity and mortality.
When compared to single-chamber ventricular pacing, single-chamber atrial pacing offers an advantage.	By preserving atrioventricular synchronization, the multi-chamber pacemaker resembles cardiac physiology more closely.

Modern pacemakers have several vital components, including the pulse generator, leads, and sensing circuitry. Advances in lead design have improved durability and reduced complications, although some complications still persist, such as infections near the pocket and lead placement, lead dislodgement due to repetitive motion, thromboembolism, pacemaker displacement, pneumothorax, and hematoma formation. During the initial days, devices were implanted by a thoracotomy. Nowadays, they are almost entirely placed using a transvenous technique through the subclavian vein [18]. The cephalic or subclavian vein is accessed by a small incision beneath the collarbone, often performed under local anaesthesia and with shorter recovery times. The leads are inserted into the cardiac muscle and fastened with small hooks or screws into the cardiac muscle. Afterward, the leads are connected to the pacemaker header block, and the gadget is implanted in a pre-pectoral pocket [1]. A well-programmed computer system that can communicate with the pacemaker through telemetry is called a programmer; this allows the specialist to monitor lead function and battery longevity and analyze enormous volumes of data detected by the pacemaker's built-in Holter system [1].

Pacemaker Indications

Nearly half of the patients with conduction abnormalities suffer from heart failure, and among them, the left bundle block is linked to a high mortality rate [19]. Pacing is now quite effective in correcting symptoms and prognosis but does not entirely imitate normal electrical cardiac biology. Thus, implanting a device only when needed is crucial to bypass any potential detrimental consequences of pacing. Pacing for CRT is an emerging indication for pacing that entails the placement of leads in specific chambers (right atrium and right ventricle) and the placement of a lead to pace the lateral wall of the left ventricle via the lateral vein inserted through the coronary sinus [20]. Medically refractory symptomatic individuals with class III/IV heart failure, a short QRS complex, and an ejection fraction of less than 35% are increasingly considered for a CRT pacemaker implant [1].

The two most prevalent causes for pacemaker implantation are the AV block and sick sinus syndrome (SSS), which entail an excessively sluggish heartbeat. Pacemakers either restore or regulate the cardiac electrical activity [17]. SSS is a grouping of cardiac arrhythmias that include sinoatrial block, sinus arrest, sinus bradycardia, and alternating paroxysmal atrial tachyarrhythmias along with bradycardia (tachy-brady syndrome). Patients may have lightheadedness, syncope, or dyspnea during bouts of bradycardia, whereas individuals with tachy-brady syndrome may develop atrial fibrillation [21].

Future of Pacemakers 

The pacemaker technology has advanced at an astounding rate throughout the years. Early devices provided essential pacing functions, but later versions included complex features, including rate responsiveness, dual-chamber pacing, and adaptive algorithms. These advancements enabled pacemakers to imitate the average heart rate response to physical exercise and provide patients with personalized pacing [22]. Nowadays, pacemakers can be programmed noninvasively, pacemaker generators include an X-ray code that may be seen on a chest X-ray [15], and leadless pacemakers (LPs) are also available in the market, which is a novel form of pacemaker that combines the generator and leads and has been proven to be a viable alternative to standard transvenous pacemakers. It is appropriate for complex or problematic conventional pacemaker implantation scenarios, such as subclavian vein obstruction, traditional pacemaker pocket infection, lead fracture, and multiple pacemaker replacements. LPs reduce pocket- and lead-related issues compared to standard pacemakers because they do not require pockets or leads [23]. Leadless cardiac pacing symbolizes the prospective of cardiac pacing systems, much as the shift from epicardial pacing systems to today's widespread transvenous systems [14].

The usage of devices in heart failure has gained traction and the possibility for significantly increased use of a wide range of devices soon [4]. The Randomized Evaluation of Mechanical Assistance Therapy as an Alternative in Congestive Heart Failure (REMATCH) trial has shown that mechanical blood pumps can enhance functional ability, symptoms, and survival of patients. Several assist gadgets with qualities, such as complete implantability, excellent durability, and smaller size, are presently being researched; they may enhance patient outcomes even more [7].

Despite the remarkable progress, challenges remain. Battery life, infection risks, and lead-related issues are ongoing concerns. Researchers are investigating innovative solutions, such as energy-harvesting mechanisms to extend battery life and materials to prevent infections. In addition, integrating artificial intelligence and machine learning promises to improve device performance and predictive capabilities [24].

ICDs

The pacemaker device has evolved along with technology. In 1980, the first implanted ICD was produced [15]. An ICD encompasses a battery; a capacitor to stock and distribute charges; a microprocessor with unified circuits for electrogram sensing; data gathering, storage, and therapy administration control; and a header to bridge the endocardial leads used for pacing, sensing, and defibrillation. These contents are housed in a titanium container and together are called as pulse generator. The interaction of these components results in the main elements of ICD operation, such as sensing, detecting, giving therapy, observing cardiac rhythms following shock delivery, and storing events. The sensing function in this process distinguishes the depolarization sequences of each atrial along with ventricular depolarization, and the detecting function uses an algorithm to classify the rhythm and determine whether treatment is necessary [25].

The ICDs that were used in the 1980s were solely intended to identify and abolish ventricular fibrillation by administering a high-energy shock [26]. Because those initial gadgets could not identify unstable ventricular tachycardias (VTs) that may develop into ventricular fibrillation, additional pacemakers were necessary to offer alternate bradycardia pacing, resulting in lethal synergy [25,13]. In the early 1990s, new-generation devices came into clinical use. ATP was incorporated in these devices and low-energy shocks for terminating VTs, substantial programmability, and telemetry functionalities [27]. For quick charging time and delivering high-voltage shocks, devices initially used had cylindrical aluminium electrolytic capacitors and silver vanadium pentoxide batteries [28]. Nevertheless, due to the short service time and high maintenance of these batteries, lithium-silver vanadium manganese oxide batteries are currently being utilized, extending an ICD's service life [29].

An ICD system's ability to successfully resuscitate a potentially deadly ventricular arrhythmia depends on effective detection and timely shock delivery. The ICD lead and ICD generator are vital elements of this device. The lead, for instance, is an absolute lifeline whose job is to transmit essential data about the cardiac rhythm to the ICD generator, which then delivers life-sustaining treatment as necessary. Malfunctioning of an ICD lead can result in severe outcomes, such as pacemaker failure, defibrillator failure, inappropriate shocks, and even death [5].

ICD Indications

An ICD is a medical gadget that monitors and treats irregular cardiac rhythms, including severe arrhythmias, such as VT and ventricular fibrillation. ICDs are also used as primary prevention in severe patients at risk of developing sustained ventricular arrhythmias that can potentially lead to sudden cardiac death. This includes individuals with certain cardiac complications, such as ischemic cardiomyopathy, non-ischemic cardiomyopathy, hypertrophic cardiomyopathy, and long QT syndrome. ICDs are commonly used in individuals who have a cardiac arrest or sustained ventricular arrhythmias (VT or ventricular fibrillation) that put them at high risk of recurrence [30].

In some cases of heart failure, especially if the heart's pumping function is significantly reduced (low ejection fraction), an ICD might be recommended to prevent sudden cardiac death. If a patient has experienced unexplained fainting (syncope), an ICD might be considered if a life-threatening arrhythmia is suspected as the cause. Patients who have survived a heart attack or undergone specific cardiac procedures, such as coronary artery bypass graft surgery, can also be candidates for an ICD [31].

It is important to note that the choice to implant an ICD is made based on a thorough evaluation by a cardiologist or an electrophysiologist who considers the patient's medical history, cardiac condition, risk factors, and potential benefits of the device. The guidelines and recommendations for ICD implantation can evolve, so it is essential to consult with a qualified medical professional for the most up-to-date and personalized information.

Future of ICDs

ICD leads, including many medical inventions, have experienced significant transformations: epicardial leads, which required a thoracotomy for lead installation, have given way to transvenous leads, which are simpler to implant, cheaper, and linked with lower morbidity and mortality [32]. The first-generation devices were relatively big, and numerous advancements in size and weight, arrhythmia discrimination, monitoring capabilities, battery technology, shock waveform and output, and defibrillator electrode technology were required to allow the present large-scale annual implantations [13]. Transvenous lead technological advancements, such as steroid elution, smaller diameter leads, innovative insulations, and multipolar leads, have resulted in significant therapeutic advantages for patients. Although contemporary ICD leads primarily consist of electrodes, conductors, insulation, and a fixation mechanism to secure the lead to the heart, the lead design and function vary by type or brand [5].

The initial ICDs were large, bulky, and costly. These devices necessitated open chest surgery and were inserted in the belly. These operations had a high risk of complications, such as systemic infections and post-operation sepsis [13]. Although many advancements have occurred in ICDs, some complications remain to be minimized, such as lead failure, generator-related infections, lead endocarditis, thrombotic events, cardiac perforations, and severe bleeding [33]. Currently, we are using a transvenous implantable cardioverter defibrillator. Still, we have another option available as subcutaneous ICDs, which have fewer complications associated with implantation and are currently used in younger patients with problematic venous access due to hemodialysis or complex heart architecture, with past device infection or at greater risk for infection. Nonetheless, the main concern is that these subcutaneous ICDs cannot be used in VT or bradycardia. It will be a significant boon if these subcutaneous ICDs function in VT or bradycardia, which is presently being researched [12].

The future of ICDs looks bright, with current research focusing on shrinking device size, increasing battery longevity, and improving lead technology. Leadless ICDs, which do not require transvenous leads, are gaining popularity because of their potential to lessen difficulties associated with lead installation. Artificial intelligence and machine learning algorithms may improve arrhythmia identification and enable personalized therapeutic changes. Telemonitoring capabilities make remote patient care, early arrhythmia diagnosis, and prompt intervention possible [9,34].

Do's and dont's for patients with a cardiac implanted electronic device

Following implantation, noninvasive regular lead evaluations should be done every three to six months to rule out the possibility of lead failure, as the lead-related problem requires surgical revision. When an ICD lead fails to administer the required high-voltage treatment, the result can be disastrous and life-threatening. Patients are more likely to present with inappropriate shocks or aberrant electrical parameters discovered after standard testing. Thus, it is essential to follow up every three to six months [5]. During follow-up, more complex information, such as information on arrhythmias and their frequency, percentage of pacing, and a variety of automatic tasks, may be retrieved from the device's memory, finally providing the patient with a steady, safe pacing system [1].

MRI scans should be avoided in places of high magnetic field due to the potential of severe problems and even fatalities induced by interactions of the magnetic resonance (MR) surrounds and the electric equipment [35]. Although MRI conditional cardiac implanted electronic devices have been commercialized in recent years, allowing patients to undergo MRI under specified conditions, they are still not widely used in practice due to their high cost, and the adaptation of this technology has been slower than expected, with conventional pacemakers accounting for the majority of implantations in many centers. Patients with implanted cardiac devices should also avoid electronic surveillance scanners, surgical diathermy [1], and dangling headphones around your neck or within 3 cm (1 in) of your ICD, keeping mobile or cordless phones, as well as MP3 players, at least 15 cm away from your ICD. Table 2 provides a summary of all articles mentioned in this review article.

**Table 2:** Summary of all articles mentioned in this review article CIEDs: cardiac implantable electrical devices; ACC/AHA/NASPE: American College of Cardiology/American Heart Association/North American Society of Pacing and Electrophysiology; ICDs: implantable cardioverter defibrillators; HF: heart failure; REMATCH: Randomized Evaluation of Mechanical Assistance for the Treatment of Congestive Heart Failure; NYHA: New York Heart Association; AV: atrioventricular; VALIANT: Valsartan In Acute Myocardial Infarction; LBBB: left bundle-branch block; MI: myocardial infarction; LV: left ventricular; ECG: electrocardiogram

Serial no.	Authors' name	Title of the article	Conclusion
1)	Toogood G. [1]	Pacemaker therapies in cardiology	Devices have progressed from simple single-chamber devices to multi-chamber devices capable of treating bradycardia, tachycardia, and heart failure. The existence of proven symptomatic bradycardia typically leads to the decision to implant a pacemaker. The most common indications are for sick sinus syndrome and heart block.
2)	Abi-Samra F [2]	Cardiac implantable electrical devices: bioethics and management issues near the end of life	When death from any cause appears impending, CIEDs may complicate dying. Under these conditions, careful consideration should be given to turning off some or all of the complex gadgets' functionalities.
3)	Gregoratos G et al. [3]	2002 ACC/AHA/NASPE Guidelines for Implantation of Cardiac Pacemakers and Antiarrhythmia Devices: summary article	The ACC/AHA/NASPE Guidelines for Implantation of Cardiac Pacemakers and Antiarrhythmia Devices incorporate numerous essential modifications in the guidelines and the supporting narrative. All new or revised recommendations are listed in the article.
4)	Boehmer J [4]	Device therapy for heart failure	Pacemakers and implanted cardioverter defibrillators (ICDs) are used to treat abnormal cardiac rhythms in a wide variety of heart disease patients, but they now have a specific position in HF care with the preventative use of ICDs in patients with severe systolic dysfunction.
5)	Maisel W et al. [5]	Implantable cardioverter defibrillator lead performance	ICDs have been shown in clinical trials to enhance survival in people at risk of sudden cardiac death. Although ICD leads are a mature technology, monitoring these devices is crucial in informing clinicians and patients about device performance and identifying malfunctioning goods as early as feasible.
6)	Addetia K et al. [6]	Cardiac implantable electronic device lead-induced tricuspid regurgitation	These devices can cause tricuspid regurgitation, as device displacement is a common complication. Various modes of treatment for these device-induced regurgitation are discussed in this article.
7)	Rose E et al. [7]	The REMATCH trial: rationale, design, and end points	Left ventricular assist devices improves the survival and quality of life of patients. This will also provide important information on device reliability and on the long-term safety profile of the device.
8)	Lee J et al. [8]	Leadless pacemaker: performance and complications	Leadless pacemakers are reliable and safe to use.
9)	Rav Acha M et al. [9]	Cardiac implantable electronic miniaturized and micro devices	The introduction of innovative communication technology enables intracardiac inter-device communication that is quick and reliable, with low interference from cardiac motion and external signals. These miniaturised leadless devices avoid most endovascular difficulties associated with conventional pacing devices, which are directly implanted into the heart.
10)	Elmqvist R [10]	Review of early pacemaker development	Over a period, pacemaker development occurs, and it gradually advances with time.
11)	Beck H et al. [11]	50th anniversary of the first successful permanent pacemaker implantation in the United States: historical review and future directions	Describes the journey of a pacemaker over a period
12)	Nicholas J, Sana M [12]	The subcutaneous implantable cardioverter-defibrillator in review	Subcutaneous ICDs are both safe and effective. Compared to the TV-ICD, which had a high conversion rate of VT/VF following shock treatment, the rates of lead-related problems were much lower with this device.
13)	van Welsenes G et al. [13]	Improvements in 25 years of implantable cardioverter defibrillator therapy	Device advances in size and weight reduction, arrhythmia discrimination, battery technology, shock waveforms, monitoring capabilities, and novel defibrillator electrodes have led to increased utilization and patient acceptability.
14)	Miller MA et al. [14]	Leadless cardiac pacemakers: back to the future	A leadless pacing system has fewer acute and chronic complications.
15)	Puette J et al. [15]	Pacemaker	The mortality rate after pacemaker implantation ranges from 1% to 4%, with problems occurring in 4-15% of patients. The presence of renal failure, a high NYHA class, a poor ejection fraction, a low platelet count, stroke, and body mass index all influence mortality and complications.
16)	Parsonnet V [16]	Types of pacemakers	A description of different types of pacemakers is explained in the article.
17)	Dretzke J et al. [17]	Dual-chamber versus single-chamber ventricular pacemakers for sick sinus syndrome and atrioventricular block	Dual-chamber pacing, or single-chamber atrial pacing, is considered superior to single-chamber ventricular pacing in that it more closely reflects cardiac physiology by preserving AV synchronization and sinus node dominance.
18)	Hauser R et al. [18]	Clinical experience with pacemaker pulse generators and transvenous leads: an 8-year prospective multicenter study	The performance of the pulse generator is satisfactory.
19)	Stephenson K et al. [19]	Long-term outcomes of the left bundle branch block in high-risk survivors of acute myocardial infarction: the VALIANT experience	During long-term followup, new LBBB was an independent predictor of all major unfavourable cardiovascular events in post-MI survivors with LV systolic failure or HF. This easily accessible ECG measure should be considered a significant risk factor for long-term cardiovascular problems in high-risk individuals following MI.
20)	Mariani JA et al. [20]	Cardiac resynchronization therapy for heart failure	Cardiac resynchronization therapy for heart failure is safe and reliable.
21)	Semelka M et al. [21]	Sick sinus syndrome: a review	Dual-chamber pacing is the optimal pacemaker setting for sick sinus syndrome patients.
22)	Das M et al. [22]	Modern pacemakers: hope or hype?	Modern pacemakers include various modes of dual-chamber pacing, rate-response algorithms with dual sensors for optimum physiological response, cardiac resynchronization therapy (CRT), arrhythmia-prevention algorithms, anti-tachycardia pacing, and hemodynamic monitoring. Overall, modern pacemakers provide adequate clinical performance with a sufficient safety margin.
23)	Le S, et al. [23]	Challenges during leadless pacemaker implantation	The clinical use of leadless pacemakers has alleviated some of the difficulties associated with conventional pacemakers. However, as its therapeutic applicability has grown, difficulties encountered during implantation have steadily developed. Some problems can be solved using balloon dilation and double snare techniques.
24)	Madhavan M et al. [24]	Advances and future directions in cardiac pacemakers: part 2 of a 2-part series	His-bundle pacing and leadless pacing are significant modifications in methodology that may result in improved therapeutic results. Battery-free pacemakers that convert mechanical motion into useable electrical energy may affect the future.
25)	Swerdlow CD et al. [25]	Advanced ICD troubleshooting: part I	Improved ICD troubleshooting is critical for assuring optimal device operation, optimizing patient outcomes, and minimizing possible dangers.
26)	Mirowski M et al. [26]	Termination of malignant ventricular arrhythmias with an implanted automatic defibrillator in human beings.	Implanted automatic defibrillator is effective in the termination of malignant ventricular arrhythmias.
27)	Bardy G et al. [27]	Clinical experience with a tiered-therapy, multiprogrammable anti-arrhythmia device.	A multiprogrammable anti-arrhythmia device can significantly improve the treatment of patients with disabling or life-threatening ventricular arrhythmias by reducing the need for antiarrhythmic drugs, lowering the incidence of inappropriate shocks, facilitating electrophysiological evaluation, and avoiding the need for dual-device therapy.
28)	Holley L [28]	Development of device therapy for ventricular arrhythmias	In recent years, implanted cardioverter defibrillators have emerged as the treatment for ventricular arrhythmias, with smaller sizes but improved therapeutic choices.
29)	Kroll M et al. [29]	Optimizing defibrillation waveforms for ICDs	In the 1990s, the introduction of current biphasic waveforms resulted in significant advances in defibrillation effectiveness.
30)	Ghzally Y, et al. [30]	Implantable defibrillator	The use of icds in clinical settings is risk-free.
31)	Al-Dadah A et al. [31]	Implantable cardioverter defibrillators improve survival after coronary artery bypass grafting in patients with severely impaired left ventricular function.	Patients with significant LV dysfunction benefit from ICD implantation following coronary artery bypass grafting (CABG) in terms of short- and long-term survival. In the case of significant LV dysfunction and CABG surgery, prophylactic ICD implantation should be considered.
32)	Maisel W [32]	Transvenous implantable cardioverter-defibrillator leads	Creating a transvenous implanted cardioverter-defibrillator (ICD) lead is a significant step forward in arrhythmia care.
33)	Vehmeijer J et al. [33]	Implantable cardioverter defibrillators in adults with congenital heart disease: a systematic review and meta-analysis	In secondary and primary prevention, a strikingly high incidence of suitable ICD treatments was documented in adult congenital heart disease patients with an ICD.
34)	Ammannaya G [34]	Implantable cardioverter defibrillators - the past, present and future	ICDs are still evolving, and research is being conducted to refine the ever-expanding indications for their usage. It is intended that as time passes, these gadgets will become more cost-effective and safer.
35)	Nordbeck P et al. [35]	Magnetic resonance imaging safety in pacemaker and implantable cardioverter defibrillator patients: how far have we come?	Magnetic resonance imaging has traditionally been considered a general contraindication in patients with cardiovascular implanted electronic devices. However, studies over the previous decade resulted in suggestions in the 2013 ESC guidelines, in which MRI may be possible. A wide range of device systems are certified for use in the MRI environment under specified conditions, often incorporating a dedicated device-specific MRI mode.

## Conclusions

The development of pacemakers and ICDs in cardiology demonstrates the substantial impact of medical technology on improving patient outcomes and quality of life. With continuous advancements, these gadgets continue to boost the quality of life of patients suffering from cardiac rhythm abnormalities. From their humble beginnings as external equipment to the miniaturized, customized, and networked wonders of today, these technologies have transformed the landscape of cardiovascular care. ICDs have grown into highly advanced devices with clinically proven efficacy in avoiding sudden cardiac death; they can function as pulse generators and defibrillators. Worldwide recommendations categorize disorders from class I, where pacing is useful for managing symptoms and prognosis, to class III, where pacing is unnecessary and may be dangerous. The most common causes are SSS and heart block. Patients must visit routine followup consultations and take precautions against electromagnetic fields. As research and innovation continue to improve these devices, the future holds even more tremendous promise for improving the lives of cardiac patients worldwide.

## References

[1] Toogood G (2007). Pacemaker therapies in cardiology. Aust Fam Physician.

[2] Abi-Samra FM (2011). Cardiac implantable electrical devices: bioethics and management issues near the end of life. Ochsner J.

[3] Gregoratos G, Abrams J, Epstein AE (2002). ACC/AHA/NASPE 2002 guideline update for implantation of cardiac pacemakers and antiarrhythmia devices: summary article: a report of the American College of Cardiology/American Heart Association Task Force on Practice Guidelines (ACC/AHA/NASPE Committee to Update the 1998 Pacemaker Guidelines). Circulation.

[4] Boehmer JP (2003). Device therapy for heart failure. Am J Cardiol.

[5] Maisel WH, Kramer DB (2008). Implantable cardioverter-defibrillator lead performance. Circulation.

[6] Addetia K, Harb SC, Hahn RT, Kapadia S, Lang RM (2019). Cardiac implantable electronic device lead-induced tricuspid regurgitation. JACC Cardiovasc Imaging.

[7] Rose EA, Moskowitz AJ, Packer M (1999). The REMATCH trial: rationale, design, and end points. The Ann Thorac Surg.

[8] Lee JZ, Mulpuru SK, Shen WK (2018). Leadless pacemaker: performance and complications. Trends Cardiovasc Med.

[9] Rav Acha M, Soifer E, Hasin T (2020). Cardiac implantable electronic miniaturized and micro devices. Micromachines (Basel).

[10] Elmqvist R (1978). Review of early pacemaker development. Pacing Clin Electrophysiol.

[11] Beck H, Boden WE, Patibandla S (2010). 50th Anniversary of the first successful permanent pacemaker implantation in the United States: historical review and future directions. Am J Cardiol.

[12] Kamp NJ, Al-Khatib SM (2023). The subcutaneous implantable cardioverter-defibrillator in review. Am Heart J.

[13] van Welsenes GH, Borleffs CJ, van Rees JB, Atary JZ, Thijssen J, van der Wall EE, Schalij MJ (2011). Improvements in 25 years of implantable cardioverter defibrillator therapy. Neth Heart J.

[14] Miller MA, Neuzil P, Dukkipati SR, Reddy VY (2015). Leadless cardiac pacemakers: back to the future. J Am Coll Cardiol.

[15] Puette JA, Malek R, Ellison MB (2023). Pacemaker. StatPearls [Internet].

[16] Parsonnet V (1969). Types of pacemakers. JAMA.

[17] Dretzke J, Toff WD, Lip GY, Raftery J, Fry-Smith A, Taylor R (2004). Dual chamber versus single chamber ventricular pacemakers for sick sinus syndrome and atrioventricular block. Cochrane Database Syst Rev.

[18] Hauser RG, Hayes DL, Kallinen LM (2007). Clinical experience with pacemaker pulse generators and transvenous leads: an 8-year prospective multicenter study. Heart Rhythm.

[19] Stephenson K, Skali H, McMurray JJ (2007). Long-term outcomes of left bundle branch block in high-risk survivors of acute myocardial infarction: the VALIANT experience. Heart Rhythm.

[20] Mariani JA, Gould PA, Broughton A, Kaye DM (2006). Cardiac resynchronisation therapy for heart failure. Intern Med J.

[21] Semelka M, Gera J, Usman S (2013). Sick sinus syndrome: a review. Am Fam Physician.

[22] DA MK, Dandamudi G, Steiner HA (2009). Modern pacemakers: hope or hype?. Pacing Clin Electrophysiol.

[23] Le S, Hua J, Kong Q, Chen Q (2023). Challenges during leadless pacemaker implantation. Cardiol Rev.

[24] Madhavan M, Mulpuru SK, McLeod CJ, Cha YM, Friedman PA (2017). Advances and future directions in cardiac pacemakers: part 2 of a 2-part series. J Am Coll Cardiol.

[25] Swerdlow CD, Friedman PA (2005). Advanced ICD troubleshooting: Part I. Pacing Clin Electrophysiol.

[26] Mirowski M, Reid PR, Mower MM (1980). Termination of malignant ventricular arrhythmias with an implanted automatic defibrillator in human beings. N Engl J Med.

[27] Bardy GH, Troutman C, Poole JE, Kudenchuk PJ, Dolack GL, Johnson G, Hofer B (1992). Clinical experience with a tiered-therapy, multiprogrammable antiarrhythmia device. Circulation.

[28] Holley LK (2007). Development of device therapy for ventricular arrhythmias. Heart Lung Circ.

[29] Kroll MW, Swerdlow CD (2007). Optimizing defibrillation waveforms for ICDs. J Interv Card Electrophysiol.

[30] Ghzally Y, Mahajan K (2023). Implantable defibrillator. StatPearls [Internet].

[31] Al-Dadah AS, Voeller RK, Rahgozar P (2007). Implantable cardioverter-defibrillators improve survival after coronary artery bypass grafting in patients with severely impaired left ventricular function. J Cardiothorac Surg.

[32] Maisel WH (2007). Transvenous implantable cardioverter-defibrillator leads: the weakest link. Circulation.

[33] Vehmeijer JT, Brouwer TF, Limpens J, Knops RE, Bouma BJ, Mulder BJ, de Groot JR (2016). Implantable cardioverter-defibrillators in adults with congenital heart disease: a systematic review and meta-analysis. Eur Heart J.

[34] Ammannaya GK (2020). Implantable cardioverter defibrillators - the past, present and future. Arch Med Sci Atheroscler Dis.

[35] Nordbeck P, Ertl G, Ritter O (2015). Magnetic resonance imaging safety in pacemaker and implantable cardioverter defibrillator patients: how far have we come?. Eur Heart J.

